# Advanced Thin Film Cathodes for Lithium Ion Batteries

**DOI:** 10.34133/2020/2969510

**Published:** 2020-02-06

**Authors:** Zhimin Qi, Haiyan Wang

**Affiliations:** ^1^School of Materials Engineering, Purdue University, West Lafayette, IN 47907, USA; ^2^School of Electrical and Computer Engineering, Purdue University, West Lafayette, Indiana 47907, USA

## Abstract

Binder-free thin film cathodes have become a critical basis for advanced high-performance lithium ion batteries for lightweight device applications such as all-solid-state batteries, portable electronics, and flexible electronics. However, these thin film electrodes generally require modifications to improve the electrochemical performance. This overview summarizes the current modification approaches on thin film cathodes, where the approaches can be classified as single-phase nanostructure designs and multiphase nanocomposite designs. Recent representative advancements of different modification approaches are also highlighted. Besides, this review discusses the existing challenges regarding the thin film cathodes. The review also discusses the future research directions and needs towards future advancement in thin film cathode designs for energy storage needs in advanced portable and personal electronics.

## 1. Introduction

Lithium ion batteries have attracted great research interests in the past few decades since the first commercialized lithium ion battery demonstration by SONY in 1990 due to its unmatchable energy and power density and its applications ranging from portable electronics to hybrid/full electric vehicles [1]. Extensive research efforts have been mostly focused on cathode material modification for advanced high performance lithium ion batteries as the cathode performance limits the cell potential and capacity of current lithium ion batteries [2, 3]. For example, olivine-type LiFePO_4_ has excellent structural stability but suffers sluggish kinetics [4], the layered-type LiCoO_2_ has low thermal stability and spinel-type LiMn_2_O_4_ suffers from bad cycling performance [5–8]. Efforts have been devoted to solve these issues, such as carbon coating [9–11], reduced particle dimensions [12], elemental dopings [13], modified chemistry [14], composite design [15], and nanostructure designs [16]. However, most of these works were performed using classical thick film electrodes processed by slurry-based approaches, which contain inactive materials that decrease the energy density of the cells and complicate the fundamental studies.

To overcome the issues brought by the inactive materials in the cathodes, binder-free thin film cathodes emerged, reported by Bates et al. in 1993 using RF magnetron sputtering [17]. This approach can eliminate the weight of inactive materials, which effectively increase the energy loading in the cell. Furthermore, the binder-free cathodes can avoid the usage of toxic solvent N-methyl-2-pyrrolidone (NMP) during the electrode processing. In terms of the cathode film thickness, it has been generally reported between several nanometers to a few micrometers for thin film cathodes in order to decrease the charge diffusion length, which makes the pristine cathode materials functional even without conductive additives and enables high power applications. Thin film cathodes can also be easily adopted for all-solid-state batteries where no flammable liquid electrolyte is used and enables dedicated applications such as implanted medical devices, flexible and portable electronics, smart cards. Additionally, thin film cathodes also take advantage of the fact that they are free of inactive additives and can be designed to achieve different microstructures or crystalline structures by various thin film techniques and are easier to explore than slurry cathodes [18].

**Figure 1:**
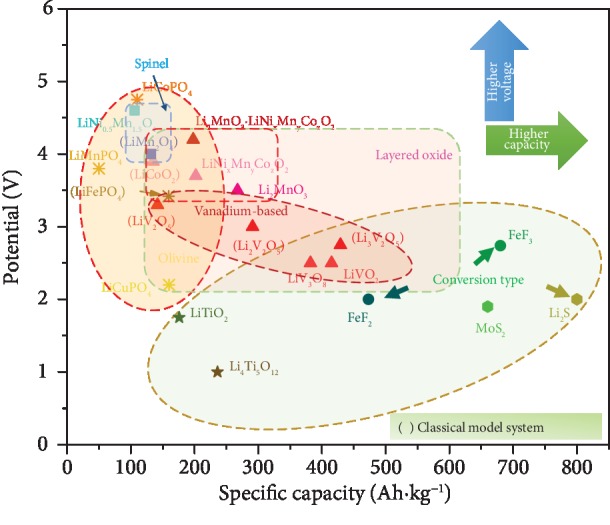
Potential versus capacity plot of the most studied cathode materials using thin film techniques.

The potential versus specific capacity plot in Figure 1 summarizes the reported up-to-date thin film cathode materials. As shown, most of the studied systems are model cathodes from different structure classes (i.e., olivine-LiFePO_4_, layered-LiCoO_2_/V_2_O_5_, and spinel-LiMn_2_O_4_). It is worth noting that several low potential materials are also plotted and are considered cathode materials due to their applications in certain 2 V batteries. In addition, layered oxides should be the more suitable choices for ideal cathode materials as they have higher voltage and higher specific capacity [19]. The review reports examples from these model cathode systems for the purpose of introducing various modification methods. The review focuses on “binder-free” cathodes using thin film processing techniques and presents several representative examples using different modification methods to demonstrate the thin film modification approaches and progresses. At the end of the review, future research directions and potentials are discussed.

## 2. Overview of Modification Approaches for Thin Film Cathodes

**Figure 2:**
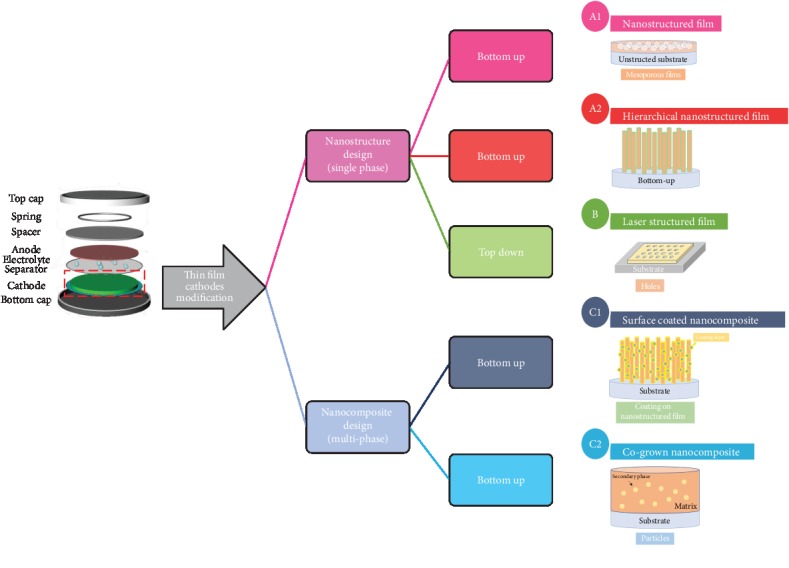
Summary of various modification approaches for cathode electrodes using thin film techniques: (A1) nanostructured cathode thin films on flat substrates; (A2) nanostructured cathode thin films on nanostructured substrates; (B) laser structured cathode thin films; (C1) cathode thin films with surface coating; (C2) multiphase nanocomposite cathode thin films.

Thin film cathode modification techniques can be categorized into two major groups, i.e., single-phase nanostructured cathodes and multiphase nanocomposite cathodes. The principal thin film techniques to achieve the abovementioned modifications include but are not limited to sol-gel method [20, 21], hydrothermal synthesis [22], coprecipitation [23], template-assisted synthesis [24], electrostatic spray deposition (EDS) [25], atomic layer deposition [26], laser processing [27], chemical vapor deposition [28], physical vapor deposition [29], plasma-assisted synthesis [30], etc. The detailed methodologies, shown in Figure 2, can be further divided as follows: (A1) to design nanostructured thin film cathodes on planar substrates with different morphologies to increase surface area; (A2) to design hierarchical nanostructured films on prenanostructured substrates where the surface areas can be further increased than the simple nanostructured thin films on planar substrates; (B) to apply laser structuring technique for posttreatment on grown thin film cathodes to achieve advanced nanostructures for larger surface area and better performance; (C1) to apply surface coatings on the grown thin film cathodes, which can also be combined with nanostructured thin film electrodes; and (C2) to achieve cogrown nanocomposite thin film cathodes for property compensation where the morphology of the secondary phase can be controlled for various designed performance.

## 3. Thin Film Electrode Modifications

### 3.1. Nanostructure Design (Single Phase)

Despite the advantages of the additive-free thin film cathodes mentioned above [31], the thin film cathodes have limited energy loading due to constraint electrodes footprint [32]. In order to solve this issue, it is necessary to increase the surface area of electrodes and harvest from 3D structures instead of planar 2D structures, which can increase the loading of active materials as well as the kinetics of electrodes [33]. Different modification approaches to achieve such a goal are introduced and discussed below.

#### 3.1.1. Nanostructured Thin Film Electrodes (a Bottom-Up Method)

**Figure 3:**
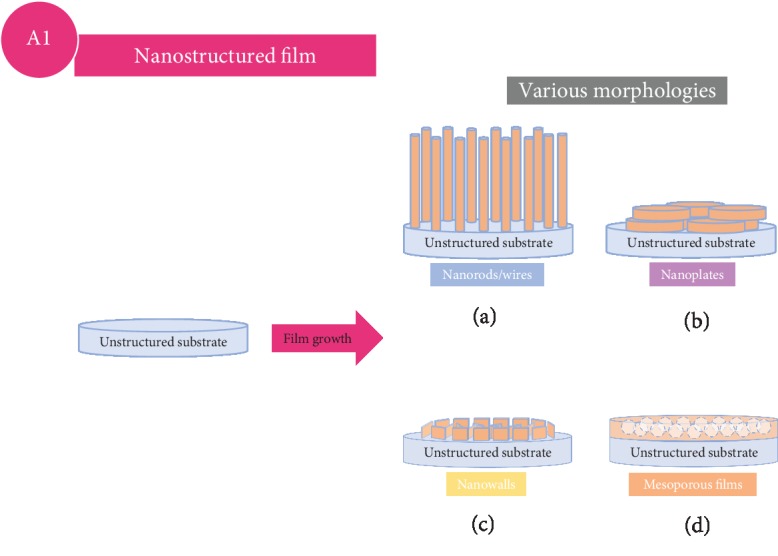
Schematic drawing of thin film modification approaches wherein nanostructured thin film cathode with different morphologies is directly grown on planar substrates.

This method refers to cathode thin films with nanostructured designs, also known as 3D cathode electrodes, on flat surface substrates. The schematic drawing is presented in Figure 3. Regarding this approach, the electrochemical performance can be tuned mainly through the different morphologies of the nanostructured designs.

*(1) Nanorod, Nanoneedle, and Nanowire Film Morphologies*. *Physical Vapor Deposition Synthesis.* Nanorod/needle-like morphology thin films of olivine-type LiFePO_4_ were reported by Sun et al. [34] using an oblique angle pulsed laser deposition (OAPLD) technique, which is simply to create a nonzero source-to-substrate angle and introduce a shadowing effect to form the isolated nanorod morphology. The method presented a slight improvement in performance compared to planar films [35]. Upon using the same OAPLD technique, Li_2_MnO_3_ thin film, a highly insulating material [36], with tilted columnar morphology has been processed and significantly improved electrochemical performance was demonstrated [37]. The film with a thickness of 150 nm was grown on Au-buffered stainless steel substrates, which gives 267.46 mAh g^−1^ initial capacity (99.5 *μ*Ah cm^−2^*μ*m^−1^) and 80% capacity retention (equal to 2 × 10^−1^% capacity loss per cycle) at 0.4 C for 100 cycles, as well as a satisfying rate performance with 147.84 mAh g^−1^ (55 *μ*Ah cm^−2^*μ*m^−1^) at 9.3 C. The electrochemical performance was improved and a higher amount of active material loading was demonstrated when compared with other reports [38–40]. The work suggests that pulsed laser deposition is an easy one-step method for introducing advanced film morphologies and complicated film compositions designs.

*Wet Chemical Synthesis.* LiCoO_2_ was explored by Xia et al. [41] using a two-step hydrothermal synthesis. This approach first grew self-supported Co_3_O_4_ nanowire arrays on Au substrates and then applied hydrothermal lithiation to achieve the lithiated LiCoO_2_ under two different temperatures, which are high temperature layered-phase LiCoO_2_ and low temperature spinel-phase LiCoO_2_ (HT-LCO and LT-LCO). The synthesized HT-LCO exhibits a hierarchical architecture where the nanowires consist of small head-to-head-connected LCO nanorods. This advanced structure shows a large areal capacity of 270 *μ*Ah cm^−2^ and a gravimetric capacity of 135 mAh g^−1^. Furthermore, HT-LCO also retains its morphology after a cycling test and keeps 90% capacity retention for 50 cycles (equivalent to 2 × 10^−1^% capacity loss per cycle) at 0.1 C. As to rate performance, it can deliver about 103 mAh g^−1^ at 10 C, which is about 76% of that at 0.1 C. This two-step hydrothermal synthesis method proposes a facile technique for 3D cathode materials that are difficult to be directly synthesized. However, the chemical lithiation process could be problematic due to the potentially time-consuming and incomplete phase transformation process. In this regard, V_2_O_5_ cathodes could be better choices due to the simplicity of synthesizing the nonlithiated phases, and many studies of growing V_2_O_5_ nanowires/nanorods thin film electrodes have been reported [42–44].

*(2) Mesoporous Film Morphologies*. Mesoporous film morphologies are widely explored as it is one of the most effective film morphologies with increased surface area and can be applied to different material systems with various thin film techniques.

*Electrostatic Spray Deposition Synthesis.* This is a widely applied technique for porous thin films as it is suitable to grow films with porous nature. During the synthesis, the precursor droplets arrive at the substrates when they are still wet and lead to simultaneous spread of droplets and evaporation of solvent, forming porous structures [45]. Furthermore, the morphologies can still be tuned by altering synthesis parameters, and it can be applied on multiple cathode systems [46–49].

*Spinel-Type Cathode Systems.* Shui et al. [50] synthesized and compared sponge-like porous, fractal-like porous, and dense LiMn_2_O_4_ thin films on Pt foils, which turned out to be the mesoporous sponge-like films with the highest surface area demonstrating the best performance with 120 mAh g^−1^ at 0.5 C and 62.4 mAh g^−1^ at 10 C. Lafont et al. [51] also studied LiNi_0.5_Mn_1.5_O_4_ and further explored the effect of solvent towards film morphologies and grain coarsening under annealing.

**Figure 4:**
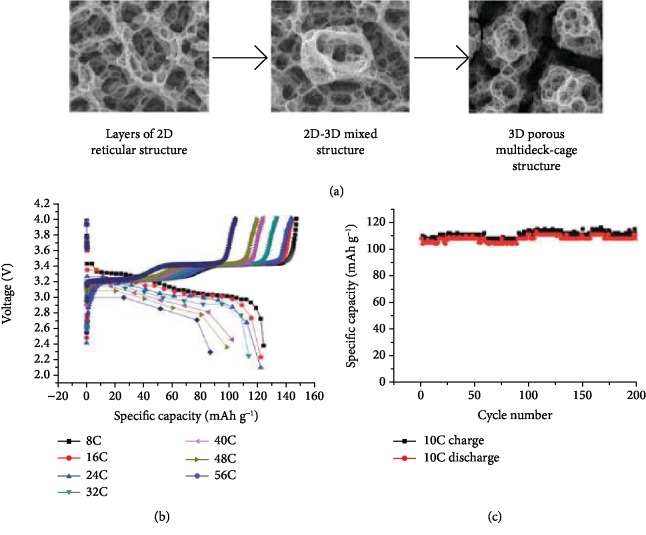
(a) The process of how the 3D porous multideck-cage structure is formed from the 2D reticular structure with increased layers. (b) Rate performance of 3D porous multideck-cage structure at 8 C, 16 C, 24 C, 32 C, 40 C, 48 C, and 56 C. (c) Cycling performance for 200 cycles at 10 C [52].

*Layered-Type Cathode Systems.* Koike and Tatsumi [53] compared porous spinel phase LT-LCO and layered phase HT-LCO and reached a similar conclusion to Xia et al.'s report [41]. The layered HT-LCO has better electrochemical performance with a capacity of 140 mAh g^−1^ at a rate of 1 C and 93% capacity retention for 100 cycles (equal to 9 × 10^−2^% capacity loss per cycle) at 1 C. Wang et al. [52] reported 3D porous V_2_O_5_ nanoparticles with “multideck cage” morphology on stainless steel substrates. As seen in Figure 4(a), confirmed by EDS, the 2D reticular structure, 2D-3D mixed structure, and 3D porous multideck cage structure were obtained sequentially simply by increasing the deposition time. This structure exhibits an excellent rate performance shown in Figure 4(b); i.e., the gravimetric capacity of the film is 142 mAh g^−1^ at 0.5 C and 86.7 mAh g^−1^ at 56 C under voltage window between 2.5 V and 4.0 V (corresponding to one Li intercalation). With respect to cycling performance, the film shows no virtual capacity loss for 200 cycles. The group also explored 3D porous Fe_0.1_V_2_O_5.15_ thin films using ESD with enhanced cyclability, suggesting the potential of obtaining complicated film compositions with controlled morphologies for cathodes using EDS.

*Template-Assisted Synthesis.* Recently, a templated sol-gel method was used to synthesize LiFePO_4_ mesoporous thin films on Pt/Si substrates [54]. Mosa et al. mixed polyisobutylene-block-poly(ethylene oxide) (PBI-*b*-PEO) amphiphilic block copolymer with LiFePO_4_ sol-gel precursors solution, which was later transferred onto substrates using dip coating to produce composite films, after which the copolymers were removed by thermal treatment to achieve the desired porous film morphology. The assembled cells show excellent electrochemical performance with a volumetric capacity of 40 *μ*Ah cm^−2^*μ*m^−1^ (gravimetric capacity of 159 mAh g^−1^) at 1.5 C and 38.5 *μ*Ah cm^−2^*μ*m^−1^ at 7.5 C. The cells can be cycled at 1.5 C with an initial gravimetric capacity of 159.43 mAh g^−1^ and 9 × 10^−4^% capacity loss per cycle, as well as a high average coulombic efficiency of 99.5%. Furthermore, the films experienced minor structural degradation despite the fact that LiFePO_4_ went through relatively large volume change during cycling [55], which is because the nanostructure is internally connected among pores, providing good structural stability [56]. Park et al. [57] also used a templated synthesis method on LiMn_2_O_4_ thin films with enhanced performance. However, the improvement is not as significant as Mosa et al.'s report on LiFePO_4_ [54]. Despite different physical properties of different cathodes, the performance could also be from the different templates used. Regarding LiFePO_4_, PBI-*b*-PEO precursors were mixed with LiFePO_4_ precursors first at an atomic level; then PBI-*b*-PEO/LiFePO_4_ composite films were deposited, whereas LiMn_2_O_4_ thin films were coated on arranged insoluble polystyrene (PS) microspheres on substrates. This indicates that synthesis methods with reactions at a finer scale should lead to better performance; for example, electrodes synthesized using the sol-gel method are expected to have better electrochemical performance than that by solid-state reactions [58].

*(3) Other Film Morphologies*. *Electrodeposition Synthesis.* Other than porous film morphologies and nanorod/pillar morphologies, several other novel film morphologies were also explored. Yu et al. [59] applied sol-gel combined anodic electrodeposition methods and grew mica-like V_2_O_5_ thin films. The electrodes exhibit a good cyclic performance with an initial capacity of 620 mAh g^−1^ (vs. Ag/AgCl) losing about 4.8 × 10^−1^% capacity per cycle for 50 cycles at 4.6 C and the morphology sustains. Xia et al. [60] applied the similar combined method for mesoporous LiMn_2_O_4_ nanowall arrays. They first deposited Mn_3_O_4_ seeds onto Au substrates using cathodic deposition techniques, then they achieved desired LiMn_2_O_4_ composition through chemical lithiation. It shows a 131.8 mAh g^−1^ gravimetric capacity at 1 C and 97.1 mAh g^−1^ at 20 C, and the cycling performance is satisfying with only 4 × 10^−2^% capacity loss per cycle for 200 cycles. This advanced 3D morphology exhibits better structural stability and electrochemical performance than the previously discussed mesoporous LiMn_2_O_4_ thin films due to more stable building blocks.

Despite various synthesis techniques or film morphologies, in general, synthesis methods that engage atomic level reactions can lead to finer structures and better performances, and morphologies with an interconnected network are usually superior in structural stability. In addition, not specified above, postdeposition thermal treatment is generally required for better electrochemical performance as it ensures crystallinity and films adhesion with substrates.

#### 3.1.2. Hierarchical Nanostructured Thin Film Electrodes (a Bottom-Up Method)

**Figure 5:**
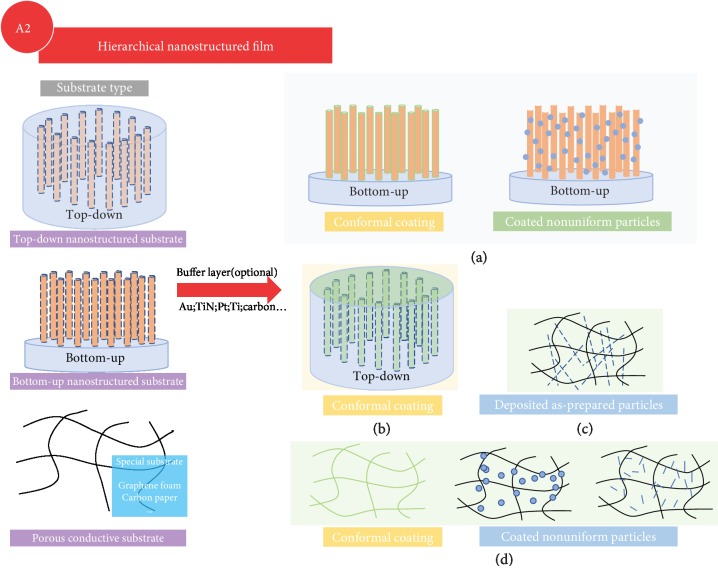
Schematic drawing of different modification approaches on nanostructured substrates: (a) nanostructured substrates obtained by bottom-up methods; (b) nanostructured substrates obtained by top-down methods; (c) direct-deposited particles on porous conductive substrates; (d) coating of cathode materials on porous conductive substrates.

Slightly different from the abovementioned section (“nanostructured film design”), this section refers to film depositions on substrates with nanostructured surfaces. As it is summarized in Figure 5, the substrates can be classified as poststructured substrates (patterned substrates) and as-prepared substrates (conductive paper/foam), and poststructured substrates can be further divided into “top-down” and “bottom-up” modified flat surface substrates. In addition to nanostructured substrates, thin films can be deposited onto the substrates surfaces through either conformal film depositions or nanostructured film depositions, where the latter is also referred to a hierarchical film architecture design. These methods usually grant more design possibilities and often provide better electrochemical performance due to better utilization of the limited substrates footprints with more sophisticated structures.

**Figure 6:**
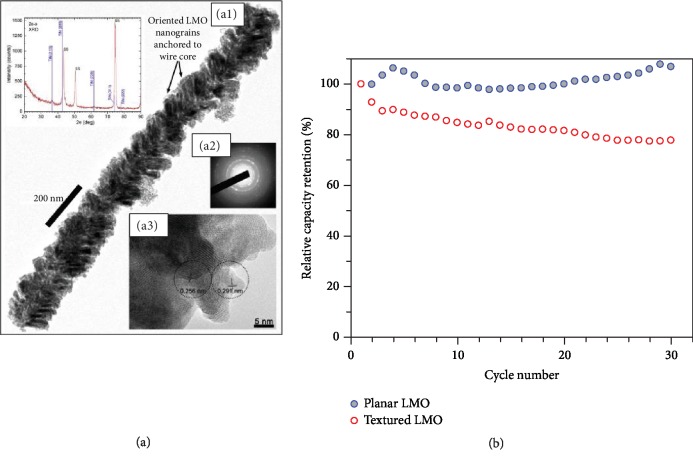
(a) TEM micrograph of individual nanowire. (b) Cycling performance of nanostructured and planar LiMn_2_O_4_ thin films for 30 cycles [61].

*(1) Nanostructured Substrates Obtained by Bottom-Up Approach*. *Plasma-Assisted Synthesis.* Bettge et al. [61] applied plasma-assisted Vapor-Liquid-Soild (VLS) methods [62] and achieved hierarchical LiMn_2_O_4_ thin film electrodes. In this work, an amorphous layer of Si was first grown on planar stainless steel (SS) substrates using DC magnetron sputtering; then the plasma-assisted VLS was realized to allow the growth of aperiodic SiO_2_ nanowires. The nanowires were then coated with metallic TiN as current collectors followed by LiMn_2_O_4_ coatings using magnetron sputtering, during which the sample stage was periodically tilted to improve film continuity. It was reported that the films contained the free-standing nanowire morphology and they were well-preserved after cycling. The detailed nanostructure of an individual nanowire is shown in Figure 6(a). It is confirmed that the LiMn_2_O_4_ thin films consist of nanocrystalline grains that are about 5 nm in size and orient around 45° with respect to the longitudinal axis of the nanowire due to the shadowing effect introduced by the oblique angle deposition. Such a morphology exhibited the nanoscale roughness with further increased surface areas (about 5 times than planar morphology), thus showing better electrochemical performance compared to planar films. However, as seen in Figure 6(b), the planar film exhibits better cyclability, which may be attributed to (1) the detachment of nanowires or LiMn_2_O_4_ grains due to the interspaced nature of nanowires and grains and (2) the Mn cation dissolution due to increased surface exposure to electrolyte [5]. Despite the undesirable cyclability, the films show an obvious decrease in charge transfer resistance due to the shortened particle dimensions as evidenced by EIS results. This work is a very comprehensive demonstration of a standard design approach especially with the readily available growth of Si nanowires on almost any substrate surfaces. However, silicon is not conductive and requires additional conductive coatings. In order to solve this issue, plasma-assisted synthesis was also applied to achieve carbon-based nanostructures [63].

*Mask-Assisted Synthesis.* Shaijumon et al. [64] grew Al nanorods directly on Al foils using pulsed potential electrodeposition with the assistance of anodic aluminum oxide (AAO) membranes [65], and LiCoO_2_ coating was obtained by thermal decomposition of spray-coated LiCoO_2_ sol-gel precursors. This method is facile for different cathode systems as Al is a suitable current collector for positive electrodes; however, the density and distribution of the Al nanorods heavily depend on the mask quality.

*Template-Assisted Synthesis.* Liu et al. [24] used engineered tobacco mosaic virus (TMV) as a template for 3D LiFePO_4_ cathodes with Ni and Ti buffer layers as current collectors. TMV is a type of cylindrical particles with a high aspect ratio and can be grafted onto metal surfaces through self-assembly. Yim et al. [66] achieved 3D hemisphere-structured LiSn_0.0125_Mn_1.975_O_4_ using PS beads as a template. A suspension of polystyrene nanoparticles was spin-coated on SiO_2_/Si substrates, which were then coated with Ti and Pt coatings, and lastly, LiSn_0.0125_Mn_1.975_O_4_ was deposited using RF sputtering. The films exhibited an increased specific capacity and rate performance, but slightly decreased capacity retention due to lack of robustness by hemisphere microstructures when compared to planar films. Template-assisted synthesis enjoys advantages regarding versatile film morphologies and material systems. However, similar to the case of the mask-assisted synthesis, the performances are heavily dependent on the templates and should be designed wisely.

*(2) Nanostructured Substrates Obtained by Top-Down Approaches*. *Chemical Etching Method.* Mattelaer et al. [67] derived patterned silicon micropillars using a top-down chemical etching technique for 3D vanadium oxide thin film cathodes with TiN and Pt as current collectors. Both amorphous and crystalline VO_2_ and V_2_O_5_ thin films were then conformally deposited on substrates using ALD, and their electrochemical performances were both improved compared to planar geometry. Besides, chemical etching can also achieve different surface morphologies [68] with further enhanced electrochemical performance. However, the thickness of films is usually not uniform throughout the surfaces due to the shadowing effect [51]. Therefore, techniques that can achieve conformal coatings are essential for excellent electrochemical performance, and ALD is currently the most fitted technique in this direction [26, 69, 70]. Although coating of randomly distributed particles can easily fix this issue, the loading of active materials is greatly reduced [68].

*Lithography Method.* Gerasopoulos et al. [71] combined top-down and bottom-up methods to achieve 3D V_2_O_5_ electrodes. First, Au micropillars were grown on silicon substrates using microplating and lithography. Then, a uniform layer of TMV nanorods was coated on Au micropillar surfaces through self-assembly [24]. Next, a uniform layer of Ni was coated on the TMV rods from an electroless plating bath followed by another layer of conformal V_2_O_5_ through ALD. The results were compared between the nanostructured V_2_O_5_ and hierarchical V_2_O_5_, and the latter one has much higher capacity and rate performance due to further increased surface area. This indicates that, by combining multiple thin film modification approaches, the design can enjoy structural versatilities for property design and the increased surface area can further enhance electrochemical performance as well as loading of active materials.

*(3) Porous Conductive Substrates with High Surface Area*. The above discussion has covered the design of a nanostructured surface on planar substrates and their effects on battery performance. Different from those substrates, some porous conductive substrates, e.g., nickel foams, graphene foams, and carbon papers, can be directly synthesized to build hierarchical 3D electrodes or self-supported flexible electrodes [72]. Among these substrates, the carbon-based substrates are the most studied due to the extremely lightweight and ability to deform.

*Deposited Particles.* Due to the porous nature of these substrates, as-grown cathode particles can be directly deposited using very simple deposition techniques [73]. Gittleson et al. [74] used spin-spray layer-by-layer deposition of V_2_O_5_ nanowires on porous Celgard separators for transparent energy storage. Seng et al. [75] mixed multiwall carbon nanotubes (MWCNTs) with ultralong V_2_O_5_ nanowires and formed self-supported flexible films using the simple membrane filtration technique. Zhang et al. [76] mixed reduced graphene oxide (rGO) nanosheets with ultralong V_2_O_5_ nanowires under hydrothermal treatment to form flexible films, where the rGO substrates not only increase the electronic conductivity of the films but also suppress the irreversible phase transition of V_2_O_5_ under wide voltage range (1.0-4.0 V).

*Grafted Particles.* Despite the simple synthesis procedures, this type of thin film requires large particles to be seated between the empty spaces of the porous substrates, which can lead to unsatisfying cycling performance as the weak material-to-substrate adhesion and limited loading of active materials. Therefore, particles can be grafted onto substrate surface through coating techniques to avoid this issue. Sathiya et al. [77] functionalized carbon nanotubes (CNTs) with concentrated nitric acid to introduce function groups and grafted V_2_O_5_ onto the surface through chemical reactions between function groups. The obtained films have a loading of 2 mg cm^−2^ and show 5% capacity drop for 25 cycles tested at 0.5 C between 1.5 V and 4.0 V. Chen et al. [78] synthesized MWCNT sponges using chemical vapor deposition, and V_2_O_5_ was coated as a conformal amorphous layer by ALD. It shows about 7.5% capacity drop for 25 cycles at 0.2 C but with much higher specific capacity than Sathitya et al.'s report [77]. This work largely improved the mass loading of active materials with the high density CNTs in the sponge, and the core-shell morphology can also overcome the issue of low electrical conductivity and mechanical strength of amorphous V_2_O_5_ [79]. Brown et al. [80] used scalable one-step pulsed electrodeposition technique and grew amorphous V_2_O_5_ on carbon nanofiber membranes (CNFs), which also demonstrated improved electrochemical performance [75].

Other cathode material systems were also explored. Chen et al. [81] grew a layer of densely anchored VO_2_ nanoflakes on carbon cloth substrates with hydrothermal technique, which gives an initial capacity of 289 mAh g^−1^ at 0.2 C and 126 mAh g^−1^ at 20 C. LiFePO_4_ nanoparticles were coated on graphene foams (GFs) by Li et al. [82] using a hydrothermal method, providing 164 mAh g^−1^ at 0.2 C and 114 mAh g^−1^ at 50 C. The work further demonstrated flexible all-solid-state battery application with good electrochemical performance under repeated mechanical deformation. Besides, LiFePO_4_ nanosheets/GFs cathodes synthesized by the same hydrothermal method show slightly better rate performance than LiFePO_4_ nanoparticles/GFs, which is expected as the hierarchical nanosheet structure has higher surface areas [83].

In conclusion, film deposition on nanostructured substrates (hierarchical nanostructured films) is a more advanced modification approach for thin film cathodes when compared to only nanostructured films. Particle-coated nanostructured substrates can have more designable variables such as particle morphologies, particle sizes, particle densities, and particle distribution, which on the other hand require more careful control of synthesis parameters. As to conformal coating, the critical issue comes to whether a uniform layer of coating can be achieved, most of which is accomplished by atomic layer deposition or other wet chemical-based methods [70, 84–86].

#### 3.1.3. Laser-Structured Thin Film Cathodes (a Top-Down Method)

The above sections introduced a series of modification approaches involving various thin film techniques such as wet chemistry, vapor deposition, and catalytical growth. Though the abovementioned approaches grant high tunability, desirable structures normally require a complicated synthesis route and postannealing is often time-consuming. Therefore, these advanced 3D electrodes are not cost-effective enough to be industrialized at this stage, not to mention that most of the work introduced above involves mainly model cathode materials, indicating its early stage development.

**Figure 7:**
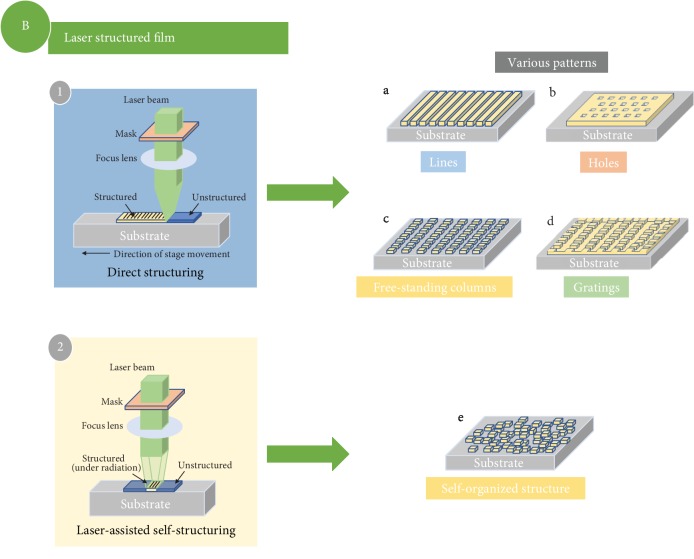
Schematic drawing of thin film modification approaches that as-deposited thin film electrodes are nanostructured using laser technique.

Compared to the above methods, laser-based cathode processing technique is a relatively new modification approach that can achieve designable 3D nanostructures on electrodes and can accomplish an annealing process on a large scale and short time [27]. This approach can also be classified as the top-down method as it is a post treatment and requires as-prepared electrodes. The basic operating principle to realize 3D electrode using laser processing techniques can be classified into two types, which are shown in Figure 7: (1) Direct structuring: a beam of laser is focused on the film surface and the stage will move in a programmable fashion. Different morphologies can be obtained by different combinations of laser mode (pulsed or continuous) and stage movement (direction and speed). (2) Laser-assisted self-structuring: laser beam is shown on the film surfaces, and the active materials will be ablated followed by subsequent redeposition on the film surface to form a structured morphology.

*(1) Direct Laser Structuring*. *Layered-Type Cathode Systems.* Most of the current works have been focused on direct structuring due to its programmable characteristics. Kohler et al. [87] used excimer source laser on RF magnetron-sputtered LiCoO_2_ thin films and obtained two different conical surfaces by changing laser operation modes. The surface structures show 5 to 10 times larger surface area than the as-deposited films. Laser annealing can be performed for only 13.2 s in ambient air at 700°C to obtain HT-LCO phase with improved crystallinity and increased grain size. Laser sources with different wavelengths are affected differently on electrochemical performance as different ablation rates result in different cone heights. This work demonstrated that the surface area can be largely increased by laser structuring, and microstructures as well as electrochemical performance can be tuned by laser operation parameters.

**Figure 8:**
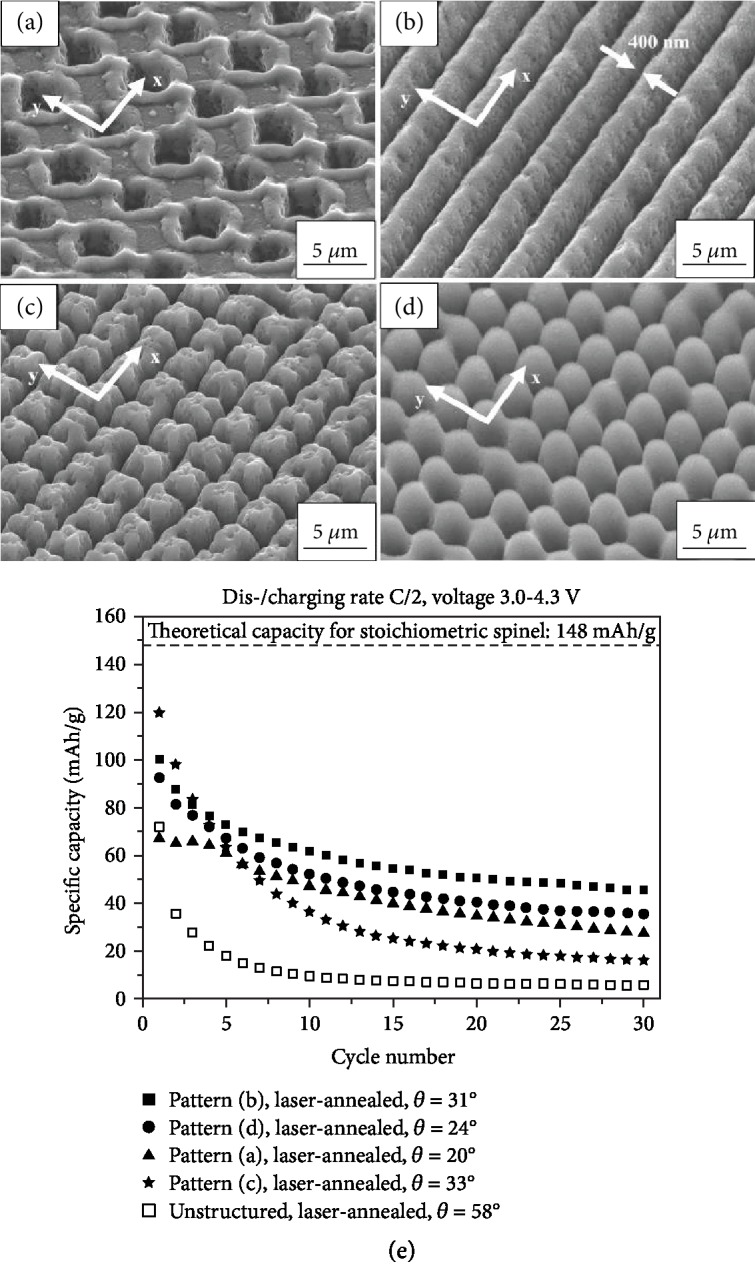
SEM micrographs with different patterns of (a) holes, (b) line, (c) grating, and (d) free-standing surface structures. (e) Cycling performance of LiMn_2_O_4_ thin films with different surface morphologies for 30 cycles at 0.5 C [88].

*Spinel-Type Cathode Systems.* Different film morphologies obtained through direct laser structuring were studied and compared. Pröll et al. [88] used LiMn_2_O_4_ thin film cathodes on stainless steel obtained by RF magnetron sputtering. The wettability of electrolyte and electrochemical performance between different surface morphologies were compared. The four different types of surface morphologies are shown in Figures 8(a)–8(d), and all the nanostructured surfaces show increased wettability with electrolyte. The cycling performance can be seen in Figure 8(e), and the grating structure (Figure 8(d)) shows the highest initial capacity of 120 mAh g^−1^ at 0.5 C but decays to only 20 mAh g^−1^ at the 30^th^ cycle. The line structure (Figure 8(b)) shows the best cycling performance while not the highest capacity, which is possibly due to the stable structure but smaller surface area when compared to the grating structure. Despite the improved electrochemical performance, the authors claim the laser-processed film contains a 30% loss of active materials due to the laser ablation, which could be a significant issue to active materials loading.

Pröll et al. [89] further optimized processing parameters for laser structuring and annealing, which lowered the active material loss to below 0.13%. However, this study shows different results from the previous report. The free-standing morphology in this study has better rate performance and cyclability compared to line structure, while the two have similar initial capacity. It was explored that the combination of laser structuring as well as laser annealing produced a hierarchical nanoscale and microscale structures which was not discussed in the previous report [88]. The postmortem analysis shows that the line structure exhibits film delamination and cracking, causing bad cycling performance. Furthermore, this result seems to be contradictory to their previous report as well [88]. Further efforts are needed in this area to reach consensus in the field.

*(2) Laser-Assisted Self-Structuring*. The obvious difference of laser-assisted self-structuring from the direct laser structuring is its free redeposition of the ablated materials on top of the pristine surface structures. Kohler et al. studied electrochemical performance of self-organized LiCoO_2_ thin film and formation mechanism of conical surfaces [90]. The film demonstrated a similar conical structure with slightly higher randomness compared to directly structured surface and an increase in electrochemical performance as well as cycling stability. Further, as stated above, the formation of such conical structures was proved to consist of different domains with different chemical compositions, thicknesses, and crystallinities, which are related to in situ grain growth from laser radiation and ex situ grain growth from redeposition of ablated particles. By applying the same principle, Hudaya et al. [91] successfully obtained 3D HT-LCO with largely enhanced rate capability.

In short, laser structuring can be a scalable and cost-effective process, and the nanostructures can be tuned by different laser sources (excimer lasers, ns fiber lasers, and fs-lasers) and operation details [92]. However, not much work is performed in the thin film electrode field despite the fact that there is an obvious need in the thin film cathode field to achieve scalable capability.

### 3.2. Nanocomposite Design (Multiple Phases)

The above sections have summarized the improvement of electrochemical performance of single phase thin film cathodes via nanostructure designs, with a basic principle, i.e., to design nanostructures with high surface areas that are mechanically stable. However, such designs can have certain inevitable issues; for example, the highly reactive surface states can favor undesirable side reactions at the electrode-electrolyte interfaces [47, 64, 77, 93]. Besides, the nanostructured materials can still experience intrinsic property issues such as low conductivity and bad chemical stability due to the limitations of selected active materials. Therefore, the concept of nanocomposite can be introduced to overcome these issues by introducing a second phase (or more) that is high in conductivity or chemical stability [94]. This is an important modification approach as it provides flexible nanostructured design approaches.

#### 3.2.1. Surface Coating Nanocomposite (a Bottom-Up Method)

**Figure 9:**
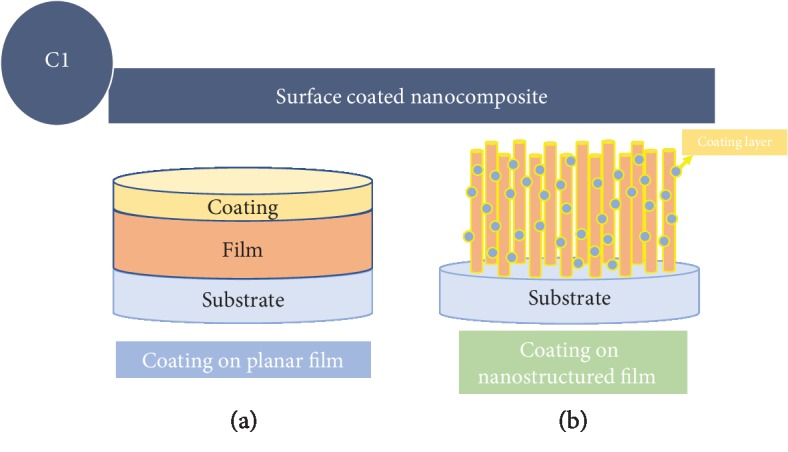
Schematic drawing of thin film modification approach of surface coating on (a) planar or (b) nanostructured thin film electrodes.

*(1) Surface Coating on Planar Thin Film Cathodes*. Nanocomposite modification types can be simply classified into two groups, i.e., introducing the second phase on top of the matrix phase, that is, surface coating. The schematic drawing in Figure 9 of different surface coating modification types can be divided into (1) surface coating on planar films and (2) surface coating on nanostructured films.

*A Physical Protection Layer.* Due to the well-known transition metal dissolution and electrolyte oxidation issues of LiMn_2_O_4_ [7, 95], this material usually requires surface coatings as physical protective layers [96]. Mattelaer et al. [18] studied the effect of ultrathin amorphous coating of Al_2_O_3_ and TiO_2_ by ALD on Pt/TiN/SiO_2_/Si substrates. Upon overcharging (>4.4 V) at a low cycle rate (0.5 C), both Al_2_O_3_ and TiO_2_ are effective in suppressing electrolyte oxidation, while TiO_2_ has slightly better suppression but also needs to go through an activation process before it can act as a protective layer. However, the rate performance of Al_2_O_3_-coated LiMn_2_O_4_ deteriorates very fast with increasing C-rate, even worse than uncoated LiMn_2_O_4_, whereas TiO_2_-coated LiMn_2_O_4_ shows an improved rate capability than uncoated LiMn_2_O_4_. This behavior is also observed morphologically, where the surface of cycled Al_2_O_3_-coated LiMn_2_O_4_ roughens more dramatically than that of TiO_2_-coated LiMn_2_O_4_, which is because the dielectric Al_2_O_3_ coating only defers the SEI formation but TiO_2_ suppresses the SEI formation as well as contributing to lithium ion conductivity. In the meantime, Teranishi et al. [97] obtained similar yet more comprehensive conclusions using LiCoO_2_ electrodes with dielectric BaTiO_3_ (BTO) coating. Fully covered planar BTO coating and dot BTO partially coated on LCO electrodes were compared, where the planar BTO shows similar results with Mattelaer case [18], but the dot BTO coating shows highly enhanced rate performance, which is explained by Teranishi et al. that the BTO-LCO-electrolyte triple junction generates negative charges by the intensified electric dipole moment at the triple junction, which attracts Li^+^ ions. This work makes a great demonstration on how thin film systems are helpful in analyzing working mechanisms of complicated nanostructured/nanocomposite electrodes without hindering effects from binders or conductive additives.

Besides electrochemically inactive coatings, researchers also applied solid-state electrolyte materials directly as coatings, such as LiPON [98] and Li_3_PO_4_ [99], to improve the electrochemical performance of thin film cathodes.

*(2) Surface Coating on Nanostructured Thin Film Cathodes*. *A Better Conductive Medium.* Surface coating techniques are usually applied together with 3D nanostructured electrodes for further improvement in performance as advanced electrodes. Olivine LiFePO_4_ cathodes are known for sluggish kinetics due to the one-dimensional lithium conduction paths and low electrical conductivity of the crystal structure [4]; surface coatings are commonly applied as better electron/ion-conducting media [9, 100]. Carbon coating was applied to tobacco mosaic virus (TMV) template-assisted LiFePO_4_ nanoforest with Ti and Ni as current collectors [24]. C@LiFePO_4_ nanoforest cathode shows much better rate performance and cycling stability with nearly 100% columbic efficiency and pertained morphology after 450 cycles. Similar structures can also be achieved through other synthesis approaches such as laser structuring on carbon-coated planar films [91] or simple PVD technique using composite targets [101].

Instead of the most commonly applied carbon coatings [11], N-doped carbon (N-C) coating can be used as an advanced coating material because N element was proven to be able to promote electron transfer and lower the energy barrier of lithium penetration in LIB applications [102–104]. A continuous layer of cross-linked N-C@LiFePO_4_ particles with nanopores was uniformly decorated on the carbon cloth surface by Pan et al. [105], which improves the gravimetric capacity, rate performance, cycling performance with decreased polarization, and charge transfer resistance when compared to LiFePO_4_/C slurry electrode.

**Figure 10:**
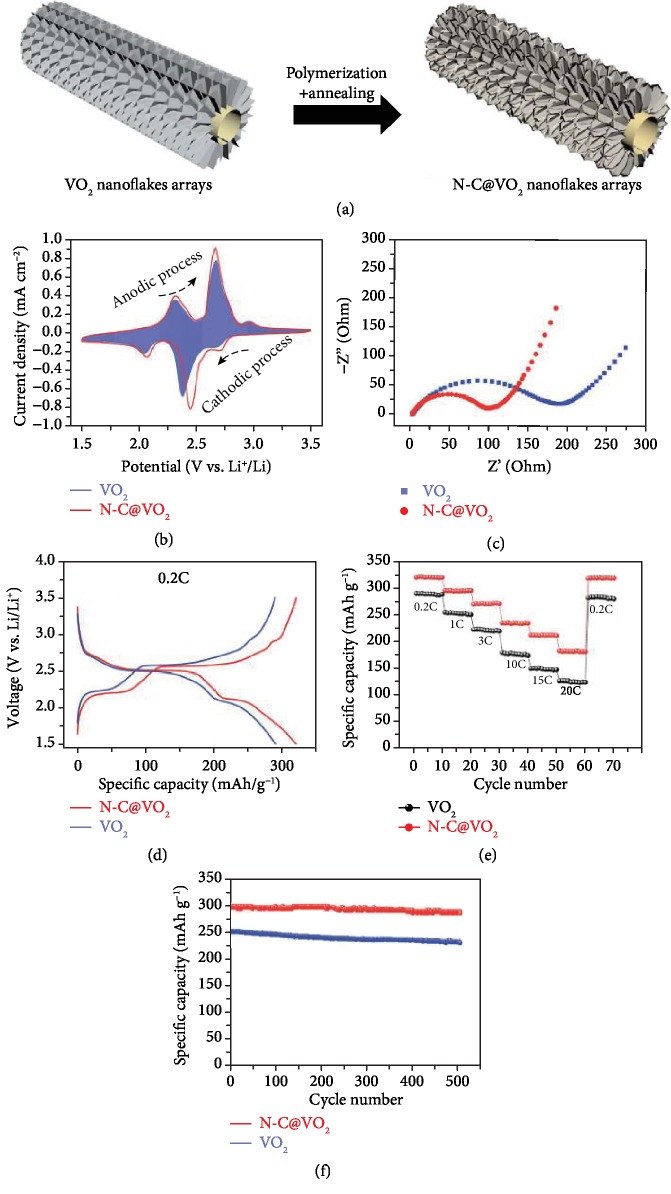
(a) Schematic illustration of the synthesis procedure of N-C@VO_2_ nanoflake arrays. (b) Cyclic voltammetry of VO_2_ nanoflake arrays and N-C@VO_2_ nanoflake arrays. (c) EIS measurements of VO_2_ nanoflake arrays and N-C@VO_2_ nanoflake arrays. (d) Charge-discharge profile of VO_2_ nanoflake arrays and N-C@VO_2_ nanoflake arrays at 0.2 C. (e) Rate performance of VO_2_ nanoflake arrays and N-C@VO_2_ nanoflake arrays at 0.2 C, 1 C, 3 C, 10 C, 15 C, and 20 C. (f) Cycling performance of VO_2_ nanoflake arrays and N-C@VO_2_ nanoflake arrays for 500 cycles at 1 C [81].

Similar to LiFePO_4_, VO_2_ also experiences low electrical conductivity. N-C coating was deposited on VO_2_ nanoflakes by Chen et al. [81] As shown in Figure 10(a), the VO_2_ nanoflakes were first anchored on carbon cloth through hydrothermal synthesis, and the N-C coating about 3 nm in thickness was subsequently coated by self-polymerization. The electrochemical performance comparison is shown in Figures 10(b)–10(f). Figure 10(b) shows that N-C@VO_2_ has better electrochemical reactivity and smaller polarization due to a better conductive network of N-C coating, which is also confirmed by the EIS results in Figure 10(c). The charge-discharge profile in Figure 10(d) of N-C@VO_2_ and VO_2_ at 0.2 C shows that N-C@VO_2_ has higher discharge capacity of 325 mAh g^−1^ at 0.2 C, lower charge plateau, and higher discharge plateau, as shown in the rate test results in Figure 10(e). Furthermore, N-C@VO_2_ showed good cycling performance with about 9.4 × 10^−3^% capacity loss per cycle at 1 C for 500 cycles.

Different from carbon-based coatings, Xia et al. [106] explored hydrogen molybdenum bronze (HMB), an n-type semiconductor with both high electrical conductivity (10^3^-10^5^ S m^−1^) and ionic conductivity (10^−3^–10^−2^ S m^−1^), as coating material to improve film kinetics. VO_2_ nanoflake arrays were obtained on graphene foams (GFs) using hydrothermal synthesis, and a layer of 15 nm thick HMB shell was coated on the nanoflakes, which largely improves the capacity and rate performance compared to similar VO_2_ nanoflakes on carbon cloth mentioned above [81]. It exhibits a gravimetric capacity of 415 mAh g^−1^ at 0.2 C and 219 mAh g^−1^ at 30 C with excellent cycling performance at very high rates (about 9.1 × 10^−3^% capacity loss per cycle at 30 C for 500 cycles).

*A HF Scavenger.* Apart from conductive coating functionality, surface coating can also act as HF scavengers [107] in liquid electrolyte-involved batteries as the inevitable trace amount of moisture in the batteries can react with electrolyte and generate HF to further attack active cathode materials and cause permanent performance decay [108]. It is worth noting that the role of surface coatings is similar to physical protective layers, but in this scenario, the side reaction specifically refers to the HF attack that only occurs in specific battery systems. Liu et al. [109] coated an amorphous layer of Li_3_PO_4_ on amorphous FePO_4_ by ALD on CNTs. The author observed the increased structural stability of Li_3_PO_4_-coated cathodes as it acts as an HF scavenger for suppressing the SEI formation. In addition, Al_2_O_3_ was coated on FeF_2_ with inverse opal Ni 3D scaffold substrates using ALD, which further confirms that combinations between 3D nanostructures and surface coating can maintain good kinetics and suppress side reactions caused by large surface areas [110].

The surface coating approach is an effective method to modify thin film cathodes, and it can be combined with nanostructured cathodes to further improve the performance. Roughly, the surface coating can act differently such as conductivity promoter [24, 81, 105, 106], protective layer [18], HF scavenger in liquid cells [107–110], or surface chemistry modifier (the last example was not introduced above) [111]. Furthermore, the surface coating morphology can be either rough particle coating, core-shell coating, or ultrathin coating with different pros and cons [111].

#### 3.2.2. Cogrown Nanocomposites (a Bottom-Up Method)

**Figure 11:**
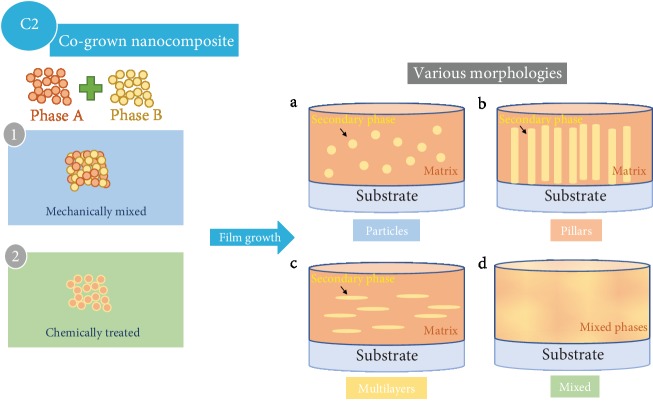
Schematic drawing of thin film modification approaches that the secondary phase and matrix phase are codeposited on substrates with tunable secondary phase morphologies. The two phases can be either mechanically mixed or chemically treated.

Apart from surface coatings, nanocomposite approaches can also be designed from the bulk perspective of thin film cathodes. The cogrown nanocomposites can be regarded as a mimic of thick film slurry electrodes with codeposition of two different phases, and the morphology of the secondary phase is tunable in the matrix phase. The most representative modification examples are summarized in Figure 11. In short, the two phases can be mixed either mechanically or chemically, which can then be cogrown on substrates with alterable morphologies such as particles, multilayers, nanopillars, or mixed domains.

*(1) Mixed Phase Nanocomposite Thin Film Electrodes*. *The Carbon Conductive Additives.* As it is introduced above, thin film electrodes generally experience slow kinetics, as most cathode materials are oxides with sluggish kinetics, and the attempt of introducing conductive materials into cathode thin films can be traced back to early 2000s. LiFePO_4_, as a model system with sluggish kinetics, was studied by Chiu [112]. He mechanically mixed LiFePO_4_ precursors with carbon sources and made LiFePO_4_/C composite targets. The LiFePO_4_/C composite films were deposited by RF magnetron sputtering on Si(100) and stainless steel substrates under optimized growth parameters, which show increased capacity and decreased resistance compared to pure LiFePO_4_. Afterward, Chiu et al. [113] further applied a layer of Ti buffer and proved it being able to enhance crystallization and grain growth and increase film-to-substrate adhesion. Further, Lu et al. [114] and Zhou et al. [115] explored the effect of carbon amount in the cathode matrix. Despite the different conclusions in detail [58], they arrived at a general trend that the specific capacity will be lowered but the kinetics of the electrodes will be improved with an increase in carbon amount. In detail, Lu et al. used poly(vinyl alcohol) and Zhou et al. applied sucrose pyrolysis as carbon sources. However, Lu et al. stated that 2 wt.% carbon content is necessary to ensure satisfying electrochemical performance and further increase the carbon amount can cause film shrinkage, delamination [114], and cracking whereas Zhou et al. claimed 23 wt.% carbon is the optimal amount and higher amount of carbon can block the lithium diffusion [115].

*The Metal Conductive Additives.* Conductive materials other than carbon were also studied. Lu et al. [116] used Ag as the secondary conductive phase. Different from the mechanically mixed carbon source and LiFePO_4_, Ag was chemically coated on LiFePO_4_ particles via a colloidal process before being mixed and pressed into a composite target, where the composite film was then deposited on Pt/Ti/SiO_2_/Si substrates using PLD. The LiFePO_4_/Ag composite films demonstrate much higher volumetric capacity, better rate performance, and increased cycling stability with a much lower weight percent of Ag compared to LiFePO_4_/C films. This behavior can be possibly explained by the more uniform mixture between active materials and conductive materials through chemical coating at an atomic level compared to simple mechanical mixing [58]. In addition to Ag, Au and mixed metals were also studied for LiFePO_4_ [117], and the mixed metal can not only increase kinetics but also prevent the active materials from unfavorable side reactions for certain cathode systems [118].

*(2) Particle-in-Matrix Nanocomposite Thin Film Electrodes*. All the above works lack the discussion from the morphological perspective of the nanocomposites; that is, no direct evidence such as TEM images was presented to confirm how the secondary phase is distributed in the matrix phase, which could lead to inaccurate conclusions. For example, Eftekhari claims that cobalt oxide exists as surface coating on LiMn_2_O_4_ through a mixed-metal codeposition process [118] because the capacity fade was suppressed and surface coating was proved to be effective in this sense [111]. However, this suppression could also be from the change of Mn valence states due to the interdiffusion of cobalt oxide phase with different morphologies [7]. This lacking perspective is critical as it can provide design insights and is helpful in studying working mechanisms.

**Figure 12:**
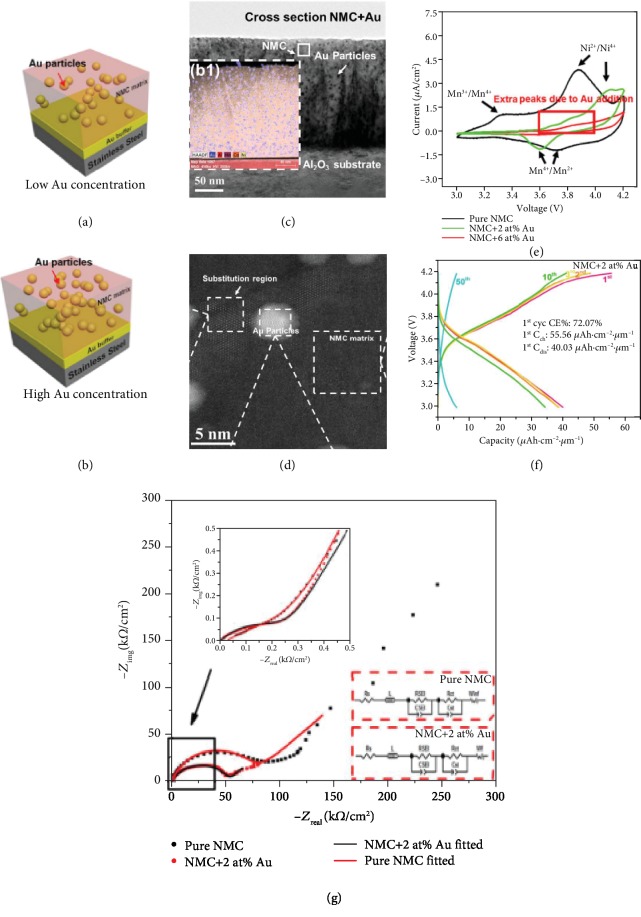
(a) Schematic drawing of NMC-Au nanocomposite with low Au concentration. (b) Schematic drawing of NMC-Au nanocomposite with high Au concentration. (c) Low-mag TEM images of cross-sectional NMC-Au nanocomposite thin film; the inset shows the corresponding EDX mapping. (d) HRSTEM of plan-view NMC-Au nanocomposite thin film. (e) Cyclic voltammetry of pure NMC, NMC-2 at% Au, and NMC-6 at% Au. (f) Cycling performance of NMC-2 at% Au nanocomposite for 50 cycles. (g) EIS results of pure NMC and NMC-2 at% Au with fitted equivalent circuits plotted [125].

*The Metal Conductive Additives.* PLD, known as an easy stoichiometry control technique [83, 119–122], combined with careful TEM studies examining the microstructures of thin film cathodes has been proven effective in studying fundamental mechanisms of cathode materials and improving electrochemical performance [37, 123–125]. Inspired by LiFePO_4_/Ag composite example, LiNi_0.5_Mn_0.3_Co_0.2_O_2_(NMC532)/Au nanocomposite thin films with different Au concentrations were deposited on Au-buffered stainless steel substrates, as schemed in Figures 12(a) and 12(b) [125]. The films were deposited by applying NMC/Au composite targets with different Au concentrations. The cross-sectional low-mag TEM images in Figure 12(c) confirm the existence of Au as embedded particles, and the inset EDX mapping confirms the correct composition of the films. Besides, the microstructure can be decided as faceted nanoparticles from the HRSTEM micrograph in Figure 12(d). It is also worth noting that some Au substituted matrix regime can be observed near domain boundaries, indicating the existence of Au dopant in NMC532. The CV results in Figure 12(e) demonstrate several facts: (1) Mn^3+^ exists in pure NMC but not NMC/Au composite films; (2) NMC/Au composites show extra Ni^2+^/Ni^4+^ peaks; (3) NMC/2 at% Au composite film has similar Ni^4+^/Ni^2+^ peak intensity but much lower Ni^2+^/Ni^4+^ peak intensity compared to pure NMC; (4) NMC/6 at% Au shows almost no intercalation reaction. The above results indicate the Au dopant has changed the chemical environment of Ni^2+^ ions and increased chemical stability. Furthermore, a moderate amount of Au addition can improve the electrochemical reversibility, but over-added Au shows almost no electrochemical performance due to the disruption of the crystal structure. The cycling performance of NMC/2 at% Au composite film for 50 cycles is shown in Figure 12(f), which is superior than pure NMC and NMC/6 at% Au (not shown here). The enhancing mechanism of Au particles can be explained with the EIS results shown in Figure 12(g), where the infinite length Warburg element is used in the equivalent circuit model to fit the data of pure NMC and finite length Warburg is used for NMC/2 at% Au composite film, which suggests the Au particles act as local current collectors in the electrode and shortens the lithium/electron diffusion pathways. What is more, the smaller semicircle of NMC/2 at% Au means a decreased charge transfer resistance due to introduction of Au particles.

*The Property Compensators.* The secondary phase can not only enhance conductivity but also act as a structural stabilizer, as a cost reducer, or simply any role to compensate for the downside of matrix materials. Shi et al. [126] designed a very simple composite structure of pure phase LiV_3_O_8_, but with nanocrystalline phases dispersed in an amorphous matrix. Amorphous LiV_3_O_8_ has larger lithium diffusion coefficient, giving higher capacity and rate performance, whereas crystalline LiV_3_O_8_ has better capacity retention. This simple nanocomposite design combined both the advantages of amorphous and crystalline LiV_3_O_8_, and the properties can be further tuned by the ratio and distribution of the two phases. Yu et al. [127] followed a similar design principle and achieved nanocrystalline Li_4_Ti_5_O_12_(LTO)/TiO_2_(TO) nanocomposite. LTO contains a stable structure upon cycling while TO has better rate performance. The nanocrystalline composite provides high density of grain boundaries and electrolyte available channels for high capacity and good cycle stability.

**Figure 13:**
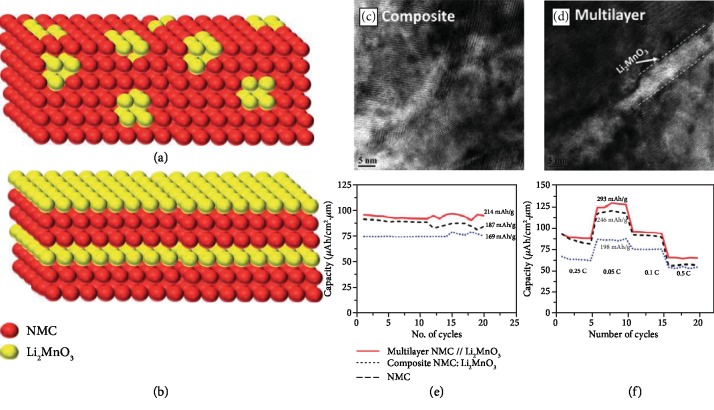
(a) Schematic drawing of composite NMC-Li_2_MnO_3_ thin film. (b) Schematic drawing of multilayered NMC-Li_2_MnO_3_ thin film. (c) TEM micrograph showing microstructure of composite NMC-Li_2_MnO_3_ thin film. (d) TEM micrograph showing microstructure of multilayered NMC-Li_2_MnO_3_ thin film. (e) Cycling performance of composite NMC-Li_2_MnO_3_, multilayered NMC-Li_2_MnO_3_, and pure NMC for 25 cycles. (f) Rate capability of composite NMC-Li_2_MnO_3_, multilayered NMC-Li_2_MnO_3_, and pure NMC. (Reproduced from Ref. [124] with permission from the Royal Society of Chemistry.)

*(3) Multilayered Nanocomposite Thin Film Electrodes*. *The Property Compensators.* The composite Li-rich cathode has a very large voltage window, high reversible capacity, and decreased cost due to the introduced secondary structural stabilizing phase (Li_2_MnO_3_), but how Li_2_MnO_3_ takes role in the composite remains debating [36]. Jacob et al. studied the performance between traditional Li-rich mixed phase composite cathode and multilayered nanocomposite where the Li_2_MnO_3_ exists in an NMC matrix with multilayer morphology [124], which is schemed in Figures 13(a) and 13(b). The multilayer composite has the isolated nanosized Li_2_MnO_3_ multilayer domains whereas the mixed phase composite has uniformly mixed two materials, as indicated in Figures 13(c) and 13(d), which allows higher capacity and better rate performance (Figure 13(f)) as the lithium diffusion behavior and pathways are more affected by NMC, that is, more conductive. In the meantime, the nanosized domains still stabilize the structure with better cycling performance shown in Figure 13(e). The work demonstrates the tunability of electrochemical performance with different morphologies of the secondary phase, and the additive-free thin films can provide new insight for nanocomposite design [128].

In short, the nanocomposite design is also a promising solution to improve the film kinetics like the single-phase nanostructure design. Additionally, with correct selection of the secondary phase, this method can help compensate for the downside of the nanostructured electrodes such as highly reactive electrode-electrolyte interfaces. However, there is lack of work regarding the morphological study of cogrown nanocomposites, which can provide insights on designing advanced thin film cathode materials.

## 4. Conclusions and Outlook

Thin film batteries are promising for high-power lithium ion batteries as the reduced thickness allows faster lithium diffusion in the electrodes. However conventional 2D planar film geometries could have limited energy loading due to the constraint footprint. Therefore, modification of thin film electrodes is necessary to meet industrial standards. In this review, we have reviewed representative advancements of thin film cathode electrodes using nanostructure and nanocomposite concepts for advanced lithium ion battery applications. It can be summarized as follows:

1. Thin film cathode materials can provide satisfying electrochemical performance without binder and conductive additives through either nanostructure or nanocomposite modification techniques
2. Among all the modification approaches for thin film cathode, nanostructure modification is well explored and studied
3. The overall design principle of nanostructure modification approaches is to increase a surface-to-volume ratio, which could, however, result in undesirable side reactions. Surface coating technique is a typical solution to prevent side reactions
4. Among the nanocomposite approaches, the coating technique is currently the most versatile and useful modification technique to prevent side reaction or increase film conductivity, and the technique of cogrowth nanocomposite has been widely utilized to improve the conductivity of thin film cathodes

In addition, several key perspectives have been summarized below:

1. Among all the techniques to achieve nanostructured thin film cathodes, electrostatic spray deposition (ESD) is a very versatile and simple technique to achieve a film with a high surface-to-volume ratio, which could be an easy choice when growing nanostructured thin film cathode
2. Substrate choice can be very versatile. Recent focus is on porous carbon-based substrates due to lightweight and high surface area, which could be a promising candidate for thin film cathode development. However, the uniform coating of the cathode is a critical factor to ensure the energy loading density and could be achieved by atomic layer deposition or some solution-based deposition techniques
3. In terms of nanocomposite design, a conclusive design principle is very much needed. For example, multiple pulsed laser deposition studies demonstrated the effectiveness of tuning the electrochemical performance of thin film cathode through controlling the morphology of the secondary phase in the matrix. A further study on the effect of secondary phase morphology towards the cathode performance is needed
4. Thin film electrodes have advantages in all-solid-state battery integration. Therefore, advanced thin film electrolyte and thin film anodes using either nanostructure or nanocomposite approach also need to be further studied. In addition, researches should be focused on interfacial interactions between electrolyte, cathode, and anode

**Figure 14:**
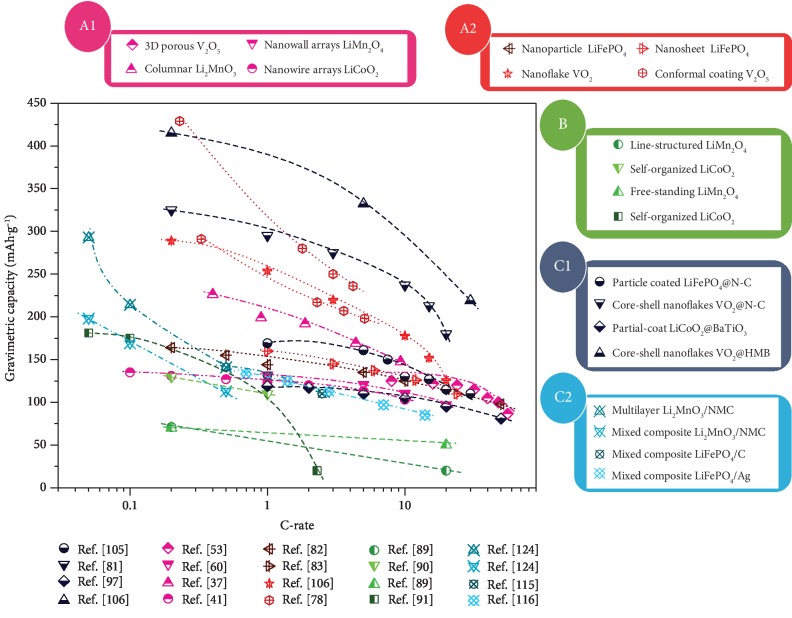
Peukert plot of representative thin film cathode electrodes modified with approaches A1, A2, B, C1, and C2 [37, 41, 53, 60, 78, 81–83, 89–91, 97, 105, 106, 115, 116, 124].

We selected the reported data of several representative modified thin film cathode materials modified by approaches A1–C2 (Figure 2) and summarized in the Peukert plot shown in Figure 14, which can provide basic guidance for future research to design advanced thin film cathode electrodes. Furthermore, we have provided the information of the thin film cathode literature about nanostructure modification and nanocomposite modification reported above in Tables 1 and 2, respectively, which summarized the cathode material system, growth method, substrate, buffer layer, and obtained morphologies. All in all, thin film cathode is a critical fundament for advanced lithium ion batteries; however, significant efforts are still required to fulfill a promising thin film cathode field with more effective modification approaches.

## Figures and Tables

**Table 1:** Summary of nanostructured thin film cathode literature discussed in this review with corresponding labeled modification approaches A1, A2, and B.

Materials	Growth method	Substrate	Buffer	Morphology	Note	Ref.
LiFePO_4_	Templated sol-gel (A1)	Si	Pt	Mesoporous	Postannealing needed	[54]
LiFePO_4_	Electrostatic spray pyrolysis (A1)	Stainless steel		Porous	Postannealing needed	[46]
LiFePO_4_	Hydrothermal (A2)	Graphene foam		Particle-coated network	Postannealing needed	[82]
LiFePO_4_	Off-axis PLD (A1)	Ti		Needle-like	Low growth temperature preferred	[34]
LiFePO_4_	Hydrothermal (A2)	Graphene foams		Nanosheet-coated network		[83]
LiFePO_4_	Coprecipitation (A2)	Graphene		Self-supported film	Flexible	[72]
VO_x_	ALD (A1)	Si	Pt/TiN	Conformal coating on Si micropillars	Crystallinity controlled by annealing	[67]
V_2_O_5_	Hydrothermal (A2)	Reduced graphene oxide nanosheet		Nanowires deposited on substrates	Postannealing needed	[76]
V_2_O_5_	Spin spray layer by layer coating (A2)	Celgard porous separator		Nanowires deposited on substrates		[74]
V_2_O_5_	Pulsed electrodeposition (A2)	Carbon nanofiber membrane		Core-shell	Postannealing needed	[80]
V_2_O_5_	Drop casting (A2)	Porous Si pillar structure	Au	Particle-coated	Postannealing needed	[68]
V_2_O_5_	Anodic deposition (A1)	Pt		Mica-like	Postannealing needed	[59]
V_2_O_5_	ESD (A1)	Pt		Porous particles	Postannealing needed	[49]
V_2_O_5_, Fe_0.1_V_2_O_5.15_	ESD (A1)	Stainless steel		Multideck cages	Postannealing needed	[52, 93]
V_2_O_5_	ALD (A2)	MWCNTs		Core-shell		[78]
VO_2_	Hydrothermal (A2)	Carbon cloth		Coated nanoflakes		[81]
LiCoO_2_	Hydrothermal (A1)	Au		Supported nanowire arrays	Chemical lithiation required; high temperature needed	[41]
LiCoO_2_	Spray coating (A2)	Patterned Al substrate		Coating on patterned substrate	Postannealing needed	[64]
LiCoO_2_	ESD (A1)	Al		Porous	Postannealing needed	[53]
LiCoO_2_	Laser printing (B)	Stainless steel		Conical structure	Predeposited film; laser annealing needed	[87]
Li_2_MnO_3_	PLD (A1)	Stainless steel	Au	Tilted columns		[37]
LiSn_0.0125_Mn_1.975_O_4_	RF sputtering (A2)	Patterned SiO2/Si	Pt/Ti	Film coated on patterned substrate	Postannealing needed	[66]
LiMn_2_O_4_	ESD (A1)	Pt		Sponge-like porous		[50]
LiMn_2_O_4_	RF sputtering (A1)	Stainless steel	TiN/SiO_2_/Si	Patterned film		[61]
LiMn_2_O_4_	Templated sol-gel (A1)	SiO_2_/Si	Pt/Ti		Postannealing needed	[57]
LiMn_2_O_4_	Laser printing (B)	Stainless steel	Au	Patterned columnar	Predeposited film; laser annealing needed	[88]
LiMn_2_O_4_	Laser printing (B)	Stainless steel		Columnar	Predeposited film; laser annealing needed	[89]
LiMn_2_O_4_	Cathodic deposition (A1)	Au		Nanowall arrays	Hydrothermal lithiation needed	[60]
TiO_2_	Catalyzed growth (A2)	Cu disk	Vertical aligned CNTs/Cr/Ni			[63]

**Table 2:** Summary of nanocomposite thin film cathode literature discussed in this review with corresponding labeled modification approaches C1 and C2.

Materials	Growth method	Substrate	Buffer	Morphology	Note	Ref.
LiFePO_4_/C	PLD (C2)	Si	Pt/Ti	Mixed nanocomposite	Postannealing needed	[114]
LiFePO_4_/C	Drop casting (C2)	Ti		Mixed nanocomposite		[115]
LiFePO_4_/Ag	PLD (C2)			Mixed nanocomposite		[116]
FePO_4_/Li_3_PO_4_	ALD (C1)	Carbon nanotubes		Core-shell	Amorphous	[109]
LiFePO_4_/N-C	Hydrothermal (C1)	Carbon cloth		Interconnected particles with porosity	Postannealing needed	[105]
VO_2_/N-C	Hydrothermal (C1)	Carbon cloth		Core-shell		[81]
VO_2_/hydrogen molybdenum bronze (HMB)	Electrodeposition (C1)	Graphene foam		Core-shell nanoflakes		[106]
LiV_3_O_8_	RF magnetron sputtering (C2)	Stainless steel		Amorphous-nanocrystalline heterostructure		[126]
LiCoO_2_/C60	RF plasma-assisted thermal evaporation then laser printing (C1)	Stainless steel	Au	Coated nanostructured film		[91]
NMC532/Au	PLD (C2)	Stainless steel	Au	Particle in matrix		[125]
NMC532/Li_2_MnO_3_	PLD (C2)	Stainless steel	Au	Multilayer in matrix		[124]
NMC532/Li_2_MnO_3_	PLD (C2)	Stainless steel	Au	Mixed nanocomposite		[124]
LiNi_0.5_Mn_1.5_O_4_/Li_3_PO_4_	ESD (C1)	Al		Coated planar film		[99]
LiMn_2_O_4_/TiO_2_	ALD (C1)	SiO_2_/Si	Pt/TiN	Coated planar film		[18]
LiMn_2_O_4_/Al_2_O_3_	ALD (C1)	SiO_2_/Si	Pt/TiN	Coated planar film		[18]
LiMn_2_O_4_/La_0.5_Sr_0.5_CoO_3_	PLD (C2)	STO		Bilayer		[128]
Li_4_Ti_5_O_12_/TiO_2_	RF sputtering (C2)	Stainless steel		Mixed nanocomposite	Postannealing needed	[127]
FeF_2_/Al_2_O_3_	ALD (C2)	Ni		Coated nanostructured film	Postannealing needed	[110]
